# Chitooligomer-Immobilized Biointerfaces with Micropatterned Geometries for Unidirectional Alignment of Myoblast Cells

**DOI:** 10.3390/biom6010012

**Published:** 2016-01-15

**Authors:** Pornthida Poosala, Takuya Kitaoka

**Affiliations:** 1 Graduate School of Bioresource and Bioenvironmental Sciences, Kyushu University, 6-10-1, Hakozaki, Higashi-ku, Fukuoka 812-8581, Japan; pin_acocyte@agr.kyushu-u.ac.jp; 2 Faculty of Agriculture, Kyushu University, 6-10-1, Hakozaki, Higashi-ku, Fukuoka 812-8581, Japan

**Keywords:** biointerface, carbohydrate-protein interaction, cell alignment, micropattern, mouse myoblast cell, oligosaccharide, tissue engineering

## Abstract

Skeletal muscle possesses a robust capacity to regenerate functional architectures with a unidirectional orientation. In this study, we successfully arranged skeletal myoblast (C2C12) cells along micropatterned gold strips on which chitohexaose was deposited via a vectorial chain immobilization approach. Hexa-*N*-acetyl-d-glucosamine (GlcNAc6) was site-selectively modified at its reducing end with thiosemicarbazide, then immobilized on a gold substrate in striped micropatterns via S–Au chemisorption. Gold micropatterns ranged from 100 to 1000 µm in width. Effects of patterning geometries on C2C12 cell alignment, morphology, and gene expression were investigated. Unidirectional alignment of C2C12 cells having GlcNAc6 receptors was clearly observed along the micropatterns. Decreasing striped pattern width increased cell attachment and proliferation, suggesting that the fixed GlcNAc6 and micropatterns impacted cell function. Possibly, interactions between nonreducing end groups of fixed GlcNAc6 and cell surface receptors initiated cellular alignment. Our technique for mimicking native tissue organization should advance applications in tissue engineering.

## 1. Introduction

Alignment is an important prerequisite for two- and three-dimensional cellular organization in various human tissues and *in vitro* cell culture applications. Cellular organization and growth behaviors enable formation of relevant constructs and are associated with physiological functions. Bioactive oligosaccharides have recently been shown to enable a wide variety of biological phenomena, such as cellular alignment, cellular recognition, cell-cell signaling, and immunological responses, through specific interactions with glyco-receptors on mammalian cell surfaces [1,2,3,4]. Such interactions, occurring at “biointerfaces”, must involve cell membranes for partitioning intra- and extracellular environments via carbohydrate-protein interactions [5,6]. Facilitating these interactions is a challenge in tissue engineering [7,8,9,10]. The mouse myoblast cell line (C2C12) represents a precursor cell in musculoskeletal myogenesis, an initial step in the formation of muscle tissues. Orientation of C2C12 cells has a strong influence on their differentiation *in vitro* into multinucleated myotubes and allows for the structural anisotropy that contributes to muscle tissue functions, such as stretching and contraction [11,12,13]. Direct regulation of cell orientation represents a crucial step towards the use of myoblast cells to develop *in vitro* applications.

However, the spatial arrangement of these cells is limited by physical factors when they are cultured on polystyrene dishes, a significant potential obstacle to promoting cellular alignment and specific biological functions. Despite various attempts to develop engineering strategies for cell alignment [14], including through mechanical loading [15], topographical patterning [16], and surface chemical treatment [17], desirable control of cell alignment has remained elusive. Reasons include inefficiencies of interactions during mature muscle formation on substrates, decreasing the potential for muscle contractility [18,19,20,21].

**Figure 1:**
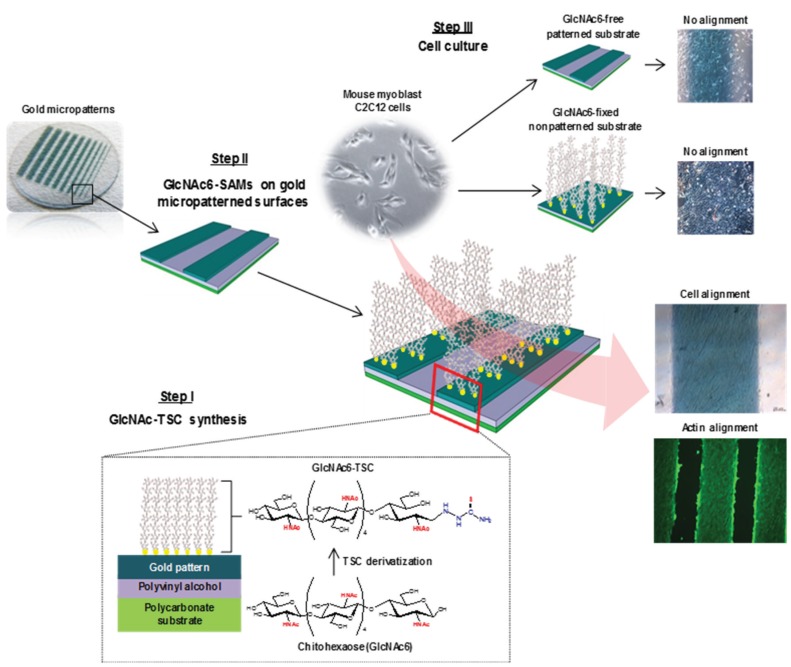
Schematic illustration of the preparation of GlcNAc6-SAMs with micropatterns and myoblast cell alignment through a specific interaction with glyco-receptors on cell surfaces.

In addition, a greater understanding of microenvironmental cues is required to drive muscle formation. Such understanding has not yet been achieved, though recent studies fabricated an aligned architecture similar to that of native muscle using synthetic polymers, such as a thermoresponsive poly(*N*-isopropylacrylamide) (PNIPAAm) [22] and soft poly(dimethylsiloxane) (PDMS) micropatterning [23]. A limitation of these materials is that they have no specific interaction with the cell surface to promote directional alignment and myogenesis regulation, both key factors for myoblast proliferation and differentiation. Furthermore, *in vivo* skeletal muscles interact with three layers of native extracellular matrix (ECM), which provides both structural support and biochemical cues that direct muscle formation. For example, Powell *et al.* created bioartificial human muscles by culturing skeletal muscle cells in a collagen/matrigel matrix before subjecting the constructs to repetitive mechanical stimulation, resulting in parallel arrangements of myofibers [24]. To mimic the highly-organized structure of skeletal muscles *in vitro*, various approaches should be investigated. In this study, we present a new approach, fabricating cell culture scaffolds that promote biological cellular organization via bioactive oligosaccharides. We previously reported various artificially-designed glyco-biointerfaces, such as self-assembled monolayers (SAMs) of chitin/chitosan, hyaluronan, cellulose and its derivatives, which were formed on gold (Au) surfaces [23,25,26,27]. The reducing end groups of carbohydrate polymers/oligomers were functionalized by a chemoselective modification with thiosemicarbazide (TSC), which enabled S–Au chemisorption to form the SAMs. The glyco-chains of the SAMs were vertically aligned on the Au surfaces when chitohexaose and cellohexaose were used [27]. This facile approach provided exposed sugar ligands (nonreducing end groups) on the substrate surface. According to our previous findings, the glyco-SAM surfaces have demonstrated desirable biocompatibility, leading to good cell adhesion and enhancing cell proliferation. The cellular activities and responsiveness to the glyco-SAMs were presumed to occur through carbohydrate-mediated specific recognition via glyco-receptors on the cell surfaces. In this work, we highlighted the successful fabrication of micropatterned SAMs of chitohexaose, composed of six *N*-acetyl-d-glucosamine (GlcNAc6) residues, on the Au-coated polycarbonate pretreated with polyvinyl alcohol (Figure 1). This served as a bioinert substrate to prevent nonspecific protein adsorption and to promote well-controlled cell alignment, representing an improved adaptation of the glyco-modification protocol established in our previous work [27]. We anticipate that the remarkable feature of the chitooligomer patterns (GlcNAc6-SAMs) will regulate fundamental cellular behaviors and may mimic the native muscle tissue environment of C2C12 cells because of the ability of specific cell surface receptors to bind to an external ligand molecule (GlcNAc6). Moreover, the intra/intermolecular hydrogen bondings of chitooligomer chains would assemble the ligands and provide unique glycoside clustering effects, which are required for cell culture engineering. Herein, we showed the influences of carbohydrate-functionalized surface geometries on the alignment and proliferation of C2C12 myoblast cells. We determined the self-assembly behavior of GlcNAc6 to form glyco-SAM architectures by quartz crystal microbalance (QCM) analysis. The biological characterizations of cultured C2C12 cells on GlcNAc6-SAMs with different geometries were investigated with regard to their initial adhesion, alignment, morphology, and early stage of cell differentiation by examining mRNA expression levels for myogenesis and bioassays.

## 2. Results and Discussion

### 2.1. Self-Assembly Immobilization of Chitooligomers on Micropatterned Gold Surfaces

Our strategy for chitohexaose-SAM (GlcNAc6-SAM) formation on Au substrates involved site-chemoselective conjugation, by which the reducing end group of GlcNAc6 was anchored to the Au surface after the TSC modification at its reducing terminus (Figure 1). This was expected to expose the carbohydrate ligands (nonreducing end groups) aligned vertically to the substrate top surfaces. The NMR spectra in Figure S1 showed the presence and absence of anomeric proton peaks at each reducing end in unmodified and TSC-modified samples, respectively. The average degree of polymerization (DP) of hydrolyzed chitooligomer, determined based on the integral ratios of internal H-1/(H-1α + H-1β), was calculated to be six, a polymer size referred to as chitohexoase. These results showed that the C1-terminal aldehyde of the GlcNAc6 pyranose ring was successfully modified with TSC to form an open ring [27]. Moreover, XPS spectra (Figure S2) revealed that the carbon elements of the GlcNAc6 residues showed three different binding energy shifts. The glyco-biointerfaces exhibited characteristic bands at 286.7 and 288.1 eV, corresponding to C–O and C=O bonds, respectively, which can be assigned to unique structures of hydroxyl C–OH and acetal (O–C–O)/acetamido (CH_3_C=O) groups in the chitooligomer molecules (Figure S2a). In contrast, the C1s spectrum of the carbohydrate-free substrates showed a weak peak at 285.0 eV, attributed to the C–C/C–H contaminants on the bare Au surface (Figure S2b). In addition, the carbon signal intensity shifted to a slightly lower binding energy because of formation of SAMs of GlcNAc6-TSC on the gold surfaces via specific thiolate anchoring [28,29]. After the appearance of the Au4f peak, the carbohydrate-SAM layer was very thin, less than 10 nm [27].

### 2.2. Surface Sugar Densities of Micropatterned Glyco-SAMs

**Figure 2:**
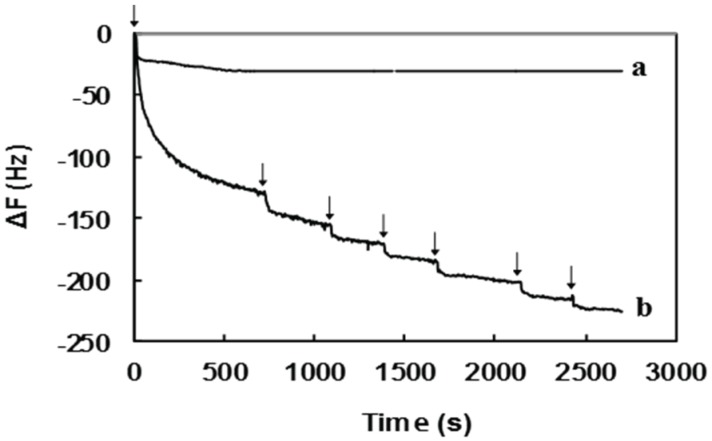
QCM profiles for spontaneous chemisorption of (**a**) TSC-free GlcNAc6 and (**b**) GlcNAc6-TSCs on a gold surface. Arrows indicate sample injection.

The chemisorption of carbohydrate-TSC molecules on the Au-coated quartz crystal of the QCM apparatus was evaluated by the steady decrease in QCM frequencies (Figure 2). After the first injection of the sugar-TSC solution, the QCM frequency first decreased rapidly and, subsequently, declined gradually upon repeated sample injections. This indicated that the carbohydrate-TSC molecules were strongly adsorbed on the Au electrode surface. The control experiment (pure GlcNAc6 without TSC) displayed a minimal significant drop in frequency after sample injections. The correlation between the change in frequencies of QCM profiles and sugar density was quantified using the Sauerbrey equation as shown in Table 1. The amount of sugar on Au patterns was 0.68 chains nm^−2^, meaning a high sugar density similar to that previously reported, 0.65 chains nm^−2^ [27], whereas TSC-free GlcNAc6 exhibited no significant adsorption. Therefore, the TSC presumably acted as an anchor, immobilizing the reducing end group of GlcNAc6 to the gold surfaces.

**Table 1:** Sugar density of GlcNAc6-TSC molecules immobilized on gold surfaces.

Sample	QCM Data		GlcNAc6 Density (Chains nm^−2^)
	∆F (Hz)	Mass (ng)	
Pure GlcNAc6	−30.7	0.92	0.09
GlcNAc6-TSC	−225.5	6.77	0.68

### 2.3. Cell Morphology and Biofunctional Behavior on Micropatterned Biointerfaces

**Figure 3:**
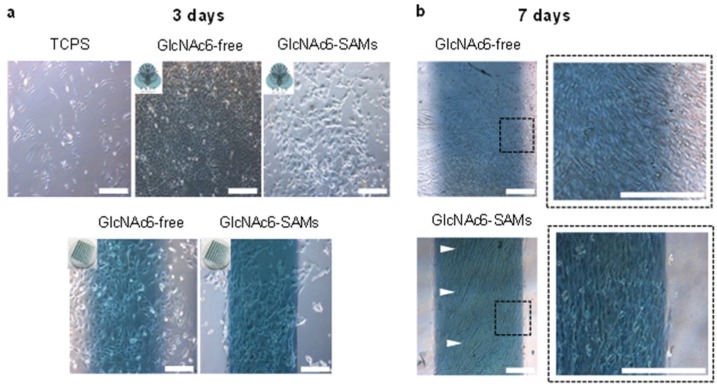
Representative phase-contrast microscopic images of mouse myoblast C2C12 cells (**a**) three days and (**b**) seven days after cell seeding on GlcNAc6-SAMs and GlcNAc6-free, with and without gold micropatterned substrates. The alignment of cultured cells was clearly dependent on GlcNAc6-SAM patterns, demonstrating a densely packed cellular assembly with the outermost cells along the edge of gold micropatterns, as shown in a dashed box. The white arrows indicated the elongated myoblasts. Scale bars correspond to 200 μm.

**Figure 4:**
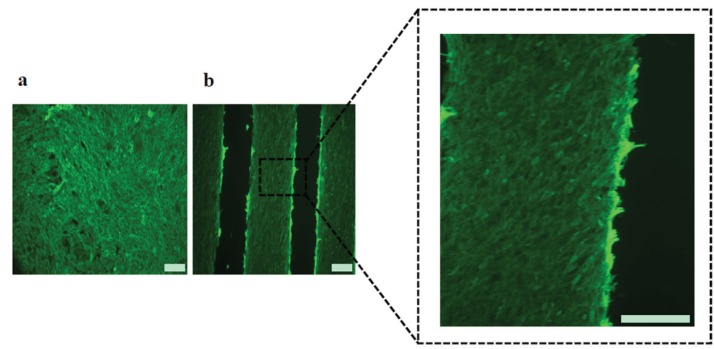
Fluorescent images showing arrangement of actin filaments after five days culture of C2C12 cells on (**a**) TCPS substrates or (**b**) micropatterned GlcNAc6-SAM. Actin filaments were stained with phalloidin (green) and the characteristic morphology of densely packed bundles of actin filaments was clearly evident in cells cultured on micropatterned GlcNAc6-SAM, as shown in a dashed box. Scale bars correspond to 200 μm.

Two different geometries, denoted as micropatterned and non-patterned GlcNAc6-SAMs, were selected for testing as functional scaffolds for engineering parallel-aligned myoblast cells, determining attachment and spreading of individual cells (Figure 3). The cells adhered to all substrates after 24 h initial seeding. Significant differences in cell morphology were observed after 3–7 days. As predicted, cells adhered on GlcNAc6-SAM micropatterns showed evidence of cell alignment, while those on the non-patterned surfaces exhibited a random orientation, indicating that the cultured cells had a flat morphology and irregular shapes on TCPS and GlcNAc6-free Au patterns (Figure 3a). Over a period of culture days, the morphology of myoblasts changed during their alignment on micropatterned GlcNAc6-SAMs, with cells becoming elongated (Figure 3b), as denoted by the white arrows. From these results, we hypothesized that the unidirectional immobilization of chitohexaose was critical to promoting cell organization, possibly through carbohydrate-protein interactions occurring during their alignment. We further proposed that alignment in response to glyco-SAMs patterns via nonreducing end groups occurred through crosstalk between adherent cells involving protein binding sites and GlcNAc receptors on the C2C12 cell surface [30,31,32]. This would suggest that cell-cell interactions play an auxiliary role as cells become confluent and can enhance cellular alignment [33]. Some research has reported that, in cell cultures, confluence is the determining factor for achieving cell alignment, though the detailed mechanisms of improving myoblast alignment through cell-cell interactions remain unknown [34]. We examined the direction of alignment of the self-organized myoblasts, appearing as highly aligned microconstructs along the edges of the striped Au surfaces of our designed GlcNAc6-SAM micropatterns (Figure 3b). Once cells began aligning on the edges, the entire micropatterned surface became covered with cells after seven days in culture. The cell bundles were densely and unidirectionally aligned on the GlcNAc6-SAM patterns. Interestingly, these alignments were similar to those formed by muscle cells *in vivo*. Another important observation was that F-actin filaments were arranged in parallel in cells cultured on GlcNAc6-SAM micropatterns (Figure 4).

This result indicated that our glyco-patterns could regulate carbohydrate-mediated signaling via F-actin anchoring of cell-substrates, potentially guiding actin alignment. The actin filament is closely associated with activation of integrin receptors at focal adhesion sites, which are important mediators of signals initiated by adhesion molecules [35,36,37]. Actin filaments are also involved in cell motility across a surface and in the contractile assemblies of muscle fibers. Existence of contractile assemblies is considered a unique property of muscle tissues. We observed preliminary evidence of actin formation aligned in parallel to the micropatterns of glyco-SAMs five days after myoblast differentiation, as indicated by fluorescent staining of F-actin with phalloidin. Actin filament formation was also observed in cells cultured on commercial polystyrene and glyco-free substrates but without any evidence of a specific cellular orientation. Here, we highlighted the possibility of direct association between bioactive oligosaccharides and cell surface receptors via carbohydrate-protein interactions, without the need for serum stimulation to enhance cell adhesion at an initial stage. Then, cells straightforwardly attached onto the GlcNAc6-SAM micropatterns and changed their morphology.

### 2.4. Effect of Micropattern Width on Gene Expression in C2C12 Cells

**Figure 5:**
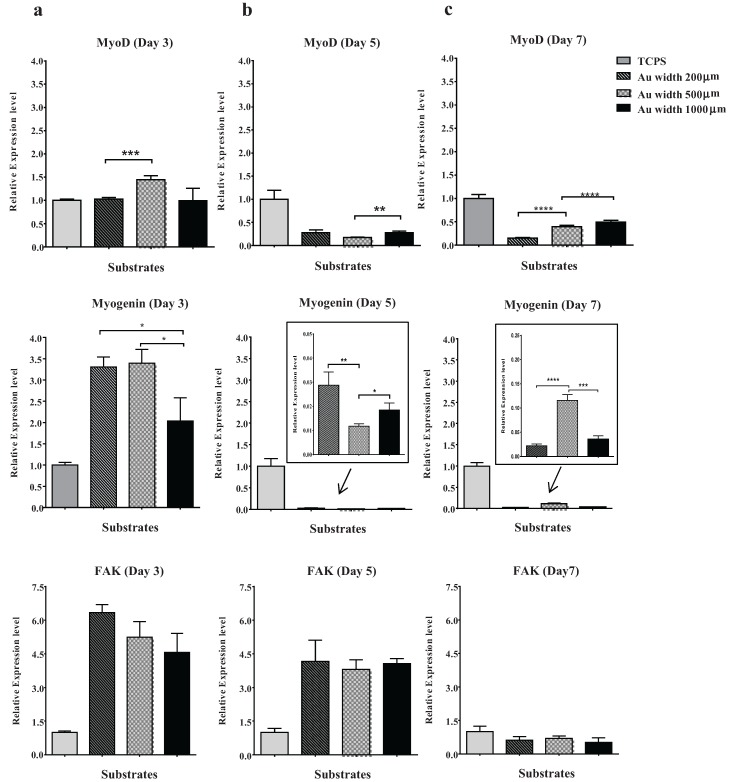
Effects of micropattern geometries on gene expression in myoblasts. RT-PCR analysis of genes associated with myogenesis of myoblast C2C12 cells, including MyoD, myogenin and FAK, on micropatterned GlcNAc6-SAMs, were observed after (**a**) three days; (**b**) five days; and (**c**) seven days of culture. Values are means ± standard error of mean. Statistically significant differences (*n* = 9 per sample); * *p* < 0.05; ** *p* < 0.01; *** *p* < 0.001 and **** *p* < 0.0001, by *t*-test.

**Figure 6:**
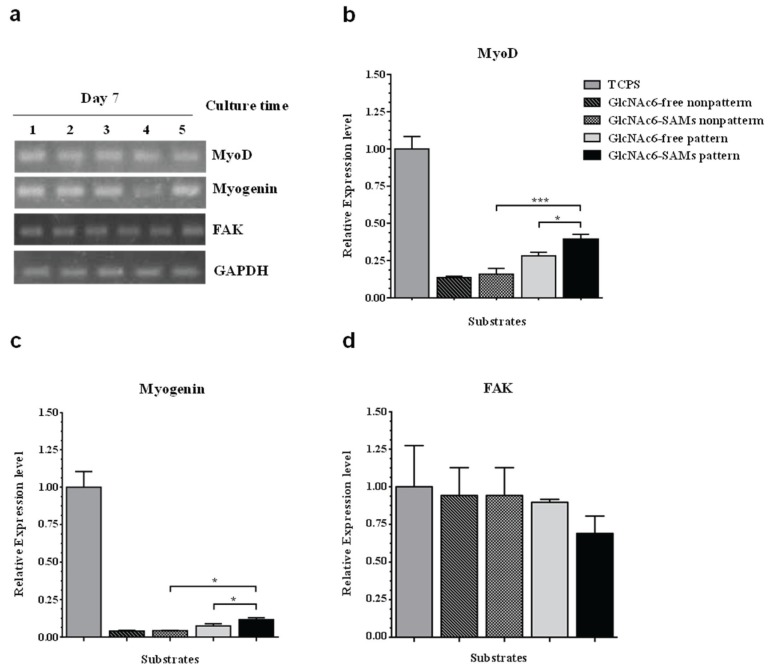
Comparison of gene expression on micropatterns with and without GlcNAc6-SAMs after seven days’ culture. The regulation of myogenin genes in myoblast C2C12 cells was detected using RT-PCR. (**a**) PCR products were analyzed on a 2% agarose gel by ethidium bromide staining. Band intensities are presented, normalized to GAPDH. Lane 1, TCPS; Lane 2, GlcNAc6-free nonpatterns; Lane 3, GlcNAc6-SAMs nonpatterns; Lane 4, GlcNAc6-free pattern (500 µm); Lane 5, GlcNAc6-SAMs pattern (500 µm); (**b**–**d**) Individual mRNA expression profiles in myoblast C2C12 cells on the different substrates.

The ability of GlcNAc6-SAM micropatterned substrates to control cell behaviors was investigated with different substrate geometries. C2C12 cells were successfully cultured and were always unidirectionally aligned on all glyco-substrates. We further observed that cells achieved the highest proliferation rates when cultured on narrower patterns, possibly because of the restricted substrate area*.* However, increasing the pattern width to 1000 µm resulted in overlapping of neighboring cells into interspacing areas between the GlcNAc6-SAM patterns. This suggested that the surface area of each pattern had a substantial effect on biological responses, such as myogenic cell differentiation. To address this possibility, we investigated mRNA expression, using RT-PCR, in cells cultured on GlcNAc6-SAM substrates with or without patterns. In this experiment, total RNA was collected from the exclusive area on GlcNAc6-SAMs to eliminate artifacts from gene expression induced by adjacent cells. The representative patterns were characterized by determining restriction patterns in widths of 200, 500, and 1000 µm. The predominant effect was a stronger expression of myoD and myogenin in C2C12 cells cultured on GlcNAc6-SAMs with certain patterns. In particular, expression of these genes after three days was higher on GlcNAc6-SAM patterns having a 500 µm width than on those on non-patterned substrates or those with widths of 200 or 1000 µm (Figure 5), possibly because of the presence of enough aligned cells to begin the differentiation. Clearly, after five days’ culture, expression of muscle regulator genes was decreased with only trace amounts of these mRNAs detectable (Figure 5b). These results were consistent with reports that the time when cultured cells have reached confluence and are ceasing proliferation represents the early stages of myogenic differentiation of myoblasts into myotubes [38,39,40]. We anticipated that such cellular alignment and drastic morphological alterations, possibly occurring through GlcNAc-mediated receptors on myoblast surfaces, might offer a desirable microenvironment to drive downregulation of myogenin via multiple intracellular signaling pathways. Such pathways are implicated in reduced transcriptional activity of the myogenin gene prior to commitment to differentiated myoblasts during terminal differentiation, though the molecular mechanisms by which myogenin controls muscle cell differentiation is still unclear. At this stage, our preliminary results may indicate that the clustering of carbohydrate oligomers had an effect not only on cell morphology and orientation, but also on dynamic activation and/or deactivation of myogenin. Notably, expression of the myogenin gene increased again after seven days in culture, possibly indicating distinct developmental stages during myogenesis. This finding was consistent with the substantial rearrangement of myoblasts and their recognition of neighboring cells following their rigorously controlled alignment on GlcNAc6-SAM patterns (Figure 5b). In contrast to that of muscle regulatory genes, FAK expression was not significantly affected by clustered carbohydrates or geometric patterns (Figure 5a–c). We believed that our GlcNAc6-SAM patterns would play an essential role in the integrin/FAK signaling pathway, which has been shown to be required for myoblast differentiation, especially for cell migration and myotube formation through cell fusion. Skeletal muscle generally expresses many integrin subunits in developmentally regulated patterns, including the integrin β1 subunit and several integrin α subunits [41,42]. Thus, we assumed that integrin activation would impact myoblast differentiation and orientation upon integrin binding to GlcNAc6 clusters. However, at some point myoblast differentiation would be indirectly inhibited by integrin due to downregulation of myoD expression. We further examined the influence of clustered carbohydrates on muscle-specific transcriptional regulation. Interestingly, expression of these targeted genes was significantly higher in cells on GlcNAc6-SAM patterns, compared with those on GlcNAc6-free patterns (Figure 6). It was reported that myoblasts on micropatterned PDMS films were well organized and were promoted to fuse along the direction of the microgrooves, but these micropatterned substrates did not significantly affect cytoskeletal markers at the transcriptional or protein levels [43]. This suggested that clustered carbohydrates more effectively facilitated cell alignment and biological responses of myoblast C2C12 cells by providing underlying topographical cues. Our novel findings represent only one of many steps needed to reach an understanding of the numerous biological signaling pathways involved in muscle regeneration. Other biomechanical aspects requiring further investigation include examining late-stage differentiation markers such as myosin heavy chain (MHC) and troponin T and evaluating contractile properties of these cellular structures by electrical stimulation. We believe that the glyco-SAMs patterns established in our study will prove useful in future research to clarify skeletal muscle development for *in vitro* applications.

## 3. Experimental Section

### 3.1. Materials

Commercially pure α-chitin powder, isolated from crab shells (Katakura Chikkarin, Tokyo, Japan), was hydrolyzed to a chitooligomer mixture and purified by the gel filtration separation (Cellufine GCL-25, JNC Corporation, Tokyo, Japan) to obtain chitohexoase (GlcNAc6). Thiosemicarbazide (TSC, Wako Pure Chemical Industries, Osaka, Japan), sodium cyanoborohydride (NaBH_3_CN, Sigma-Aldrich, St. Louis, MO, USA) and polyvinyl alcohol (PVA, MW 2000 Da, Kishida Chemical, Osaka, Japan) were used as received. The immortalized mouse muscle myoblast cell line C2C12 (ATCC-CRL1772™, ATCC, Manassas, VA, USA) was grown under standard conditions with Dulbecco’s Modified Eagle’s Medium (DMEM, Life Technologies, Tokyo, Japan) supplemented with 10% fetal bovine serum (FBS, Biowest, Nuaillé, France). Trypsin-ethylenediaminetetraacetic acid (EDTA, Invitrogen, Tokyo, Japan) solution, 0.4% trypan blue solution (Invitrogen), phosphate buffered saline (PBS, Nissui Pharmaceutical, Tokyo, Japan), phalloidin-Alexa Fluor^®^488 conjugate (Lonza, Walkersville, MD, USA), and molecular biology grade Triton^®^ X-100 (EMD Biosciences, San Diego, CA, USA) were used for biological assays. Tissue culture polystyrene (TCPS) dishes and TCPS plates (24-well) were purchased from Sumitomo Bakelite Co. Ltd. Tokyo (Japan). The water used in this study was purified with a Milli-Q system (Sartorius Stedim Biotech, Bohemia, NY, USA). Unless otherwise indicated, all chemicals were reagent grade and were used without further purification.

### 3.2. Preparation of Micropatterned Glyco-SAMs on Gold Surfaces

GlcNAc6 fractions were obtained by acid hydrolysis of chitin and gel filtration as described in Section 3.1. Purified GlcNAc6 was site-selectively modified at its reducing end group with 1 M TSC through reductive amination with 2 M NaBH_3_CN with stirring at 70 °C for 48 h, as illustrated in Figure 1. Unreacted GlcNAc6 and impurities were removed by precipitating the product in methanol, followed by centrifugation (5439 *g*, Tomy MX-301, Tokyo, Japan) at room temperature for 15 min; in this manner, the precipitate was washed seven times in methanol. GlcNAc6-TSC powder was obtained by freeze-drying the final pellet.

Polycarbonate substrates (PC; diameter 15 mm, thickness 0.5 mm) were immersed in solution (containing 1% (*w*/*v*) PVA, 5% (*v*/*v*) ethyl acetate and 20% (*v*/*v*) ethanol) at room temperature for 1 h and then air-dried. Gold micropatterns, with a range (150–1000 µm) of widths and a typical discrete width (200, 500, and 1000 µm), were ion sputtered (VPS-020, ULVAC Inc. Miyazaki, Japan, current 10 mA, 3 min, 1.5 mPa) with gold on bioinert PVA-treated transparent PC substrates using template masks (Microtech Laboratory, Kanagawa, Japan). In this manner, ultraflat (approximately 30 nm thickness) gold (Au) micropatterns were obtained. The striped Au micropatterns were immersed in a 1 M aqueous solution of GlcNAc6-TSCs, fixing polymers on the micropatterns through S–Au chemisorption and leading to the formation of SAMs on a gold surface. Preparation of micropatterned GlcNAc6-SAMs and the general procedure for cell culture on the GlcNAc6-immobilized gold micropatterns are illustrated in Figure 1.

### 3.3. Characterization of Micropatterned Glyco-SAMs

#### 3.3.1. NMR Analysis

TSC modification of carbohydrates was determined by ^1^H-NMR spectroscopy (JNM-AL 400, JEOL Ltd., Tokyo, Japan). GlcNAc6-TSC (20 mg) was dissolved in 750 µL deuterium oxide in a 5-mm capillary NMR tube. ^1^H NMR chemical shifts were expressed in ppm; a standard sodium 3-trimethylsilylpropane sulfonate (TSP) was included. The chemical shifts of the anomeric protons in chitohexoase at 5.20 and 4.55–4.65 ppm correspond to the α- and β-anomers, respectively, at each reducing end. Axial protons at internal C1 positions were assigned at 3.2–4.2 ppm [44,45]. Acetyl and ring protons appear at 2.02–1.98 and 3.74–3.92 ppm, respectively [45]. The integrated intensities of the signals were used to estimate the degree of polymerization of the chitooligomer.

#### 3.3.2. Quartz Crystal Microbalance Analysis (QCM)

Quantitation of carbohydrate-TSC molecules chemisorbed on the gold surfaces was performed with a QCM apparatus (AFFINIXQ, Initium Inc., Tokyo, Japan) with a 27 MHz AT-cut crystal resonator. The frequency changes of the sensor chip were monitored by injecting dilute aqueous solutions of GlcNAc6-TSC (1 mM) at 25 °C. The injection was repeated until the frequency reached equilibrium. The approximate amounts of chemisorbed carbohydrates on the QCM chip were calculated using Sauerbrey’s Equation (1) as described in our previous study [27]:

(1) $$\Delta F=\frac{f_{0}\Delta M\times 6.022\times 10^{23}}{\left(M_{w}\right)A}$$

In Equation (1), ΔF is the final frequency change and f_0_ is the resonant frequency. ΔM, the mass change, indicates the amount of carbohydrates adsorbed on the QCM chip (1 Hz = 30 pg). M_W_ is the molecular weight of the carbohydrate-TSC (1310.5 Da). The Au electrode surface area, A, of the quartz probe is 4.9 mm^2^.

#### 3.3.3. X-Ray Photoelectron Spectroscopy (XPS)

Elemental analysis of the carbohydrate-TSC molecules absorbed on the gold surfaces was performed with an AXIS-HSi XPS apparatus (Shimadzu/Kratos Co. Ltd., Kyoto, Japan). XPS measurements were performed at 12 kV and 10 mA with a monochromatic Al *K*α X-ray source (1486.6 eV) with the analyzing chamber pressure maintained below 0.5 µPa during the measurements. The pass energy and step width were set at 40 and 0.05 eV, respectively. The binding energies for all spectra were referenced to the C1s signal (reduced C–C band) at 285.0 eV. [46].

### 3.4. Cell Culture Assays and Microscopic Observations

C2C12 myoblast cells were cultured in DMEM supplemented with 10% (*v*/*v*) FBS. Unattached cells were removed by washing with PBS. Gold substrate was placed in a 24 well-plate and sterilized with ultraviolet light prior to cell seeding. Each cell suspension (0.5 mL, 5.0 × 10^4^ cells·mL^−1^) was seeded on each gold substrate with 0.5 mL of cell culture medium. Samples were incubated for one, three, five, and seven days at 37 °C in an atmosphere of 95% air, 5% CO_2_. Cells began to adhere within 24 h and were confluent at 7 d. The number of viable cells was measured with an automated cell counter (TC 20TM, Bio-Rad Laboratories, Inc., Philadelphia, PA, USA) after treating them with a 0.4% trypan blue solution. Cell behaviors and morphologies were observed under a phase-contrast microscope (Leica DMI 4000B microscope, Wetzlar, Germany).

### 3.5. Biological Characterization

#### 3.5.1. F-Actin Staining

Alignment of actin filaments was visualized by fluorescence staining for F-actin. Cultured cells were rinsed twice in pre-warmed PBS, fixed with 3.7% formaldehyde for 10 min and permeabilized with 0.1% Triton^®^ X-100 in PBS for 5 min. Fixed cells were pre-incubated with 1% bovine serum albumin in PBS for 20 min to block non-specific protein binding and to enhance fluorescence intensity. Filamentous actin was stained with Alexa Fluor^®^488-conjugated phalloidin according to manufacturer’s protocol. Images of stained sections were acquired using a Leica DMI 4000B microscope.

#### 3.5.2. RNA Extraction and Quantitative Real-Time PCR

Total RNA was isolated from cell preparations using ISOGEN (Nippon Gene Co., Ltd., Toyama, Japan) according to the manufacturer’s instructions. The purity and concentration of each RNA preparation was assessed using a NanoDrop™ 1000 spectrophotometer and Experion (Bio-Rad Laboratories Inc., Hercules, CA, USA), consisting of a 20 ng cDNA template, 10 µM of forward and reverse primers, and fast in 10 µL of SYBR Green Master Mix (Applied Biosystems™, Life Technologies, New York, NY, USA). The primer sequences of each gene, MyoD, Myogenin, and FAK was designed by the primer 3 plus software and blasted in the NCBI PubMed primer blast software. Forward primer: 5′ CCGTGTTCCTACCCCCAATG 3′ and reverse primer: 5′ AAGCCCAGCTCTCCCCATA 3′ for GAPDH, and forward primer: 5′ TACAGTGGCGACTCAGATGC 3′ and reverse primer: 5′ CACTGTAGTAGGCGGTGTCG 3′ for MyoD, and forward primer: 5′ GTGCCCAGTGAATGCAACTC 3′ and reverse primer: 5′ GCAGATTGTGGGCGTCTGTA 3′ for myogenin, and forward primer: 5′ ACAGACAAAGGCTGCAATC 3′, and reverse primer: 5′ GCACCAGCGATTTTGAGTTG 3′ for FAK. Amplification and quantification of mRNA were performed at 95 °C for 20 s, followed by 3 s at 95 °C, and 30 s at 60 °C for 40 cycles. After amplification, a melting curve analysis was performed to verify amplification product specificity. The relative expression levels of targeted genes were calculated and normalized by subtracting the corresponding GAPDH threshold cycle (CT) values and using the ΔΔCT comparative method [47]. PCR products were separated on 2% agarose gels, stained with ethidium bromide and photographed under UV light to confirm the single amplicon.

#### 3.5.3. Statistical Analysis

Bioassay results for individual samples were independently performed in triplicate using GraphPad Prism version 6.0 software (GraphPad Software, Inc., La Jolla, CA, USA). Data are expressed as means ± standard error of the mean (SEM). Statistical differences between two groups were evaluated using an independent samples *t*-test. Statistical significance is indicated by *p*-values of: * *p* < 0.05, ** *p* < 0.01, *** *p* < 0.001 and **** *p* < 0.0001.

## 4. Conclusions

Chitooligomer-SAM micropatterns on a transparent Au-coated polycarbonate surface were successfully fabricated by site-selective TSC-modification of chitohexaose at its reducing end, followed by S-Au chemisorption on the Au surface. Myoblast cells initially exhibited an apparently random attachment on these glyco-SAMs but gradually demonstrated unidirectional alignment along the micropatterned lines, after seven days in culture adopting an appearance similar to that of muscle cells *in vivo.* The underlying effects of micro-topographical patterns consisting of nonreducing ends of GlcNAc6 oligomers provide crucial insights into regulation of myoblast morphology and function, possibly involving carbohydrate-protein interactions. Controlled micropatterning for cell culture systems, based on our findings, represents a significant advance for future tissue engineering applications of glyco-biointerfaces.

## Supplementary Files

Supplementary File 1
