# Supplementary material for: Chitooligomer-Immobilized Biointerfaces with Micropatterned Geometries for Unidirectional Alignment of Myoblast Cells
Source: Biomolecules. 2016 Jan 15;6(1):12. doi: 10.3390/biom6010012 (PMC4808806; doi:10.3390/biom6010012)
Supplement: Supplementary File 1 [file biomolecules-06-00012-s001.pdf]

Supplementary Materials

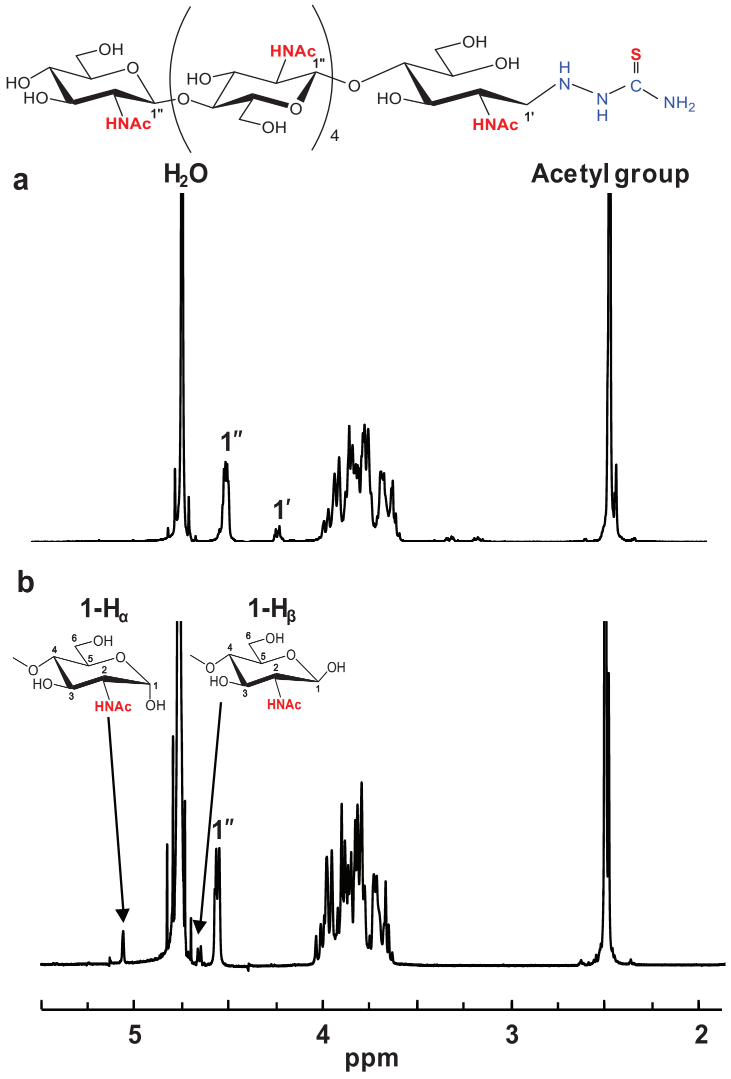

Figure S1. ^{1}H NMR spectra of GlcNAc6 with (**a**) or without (**b**) TSC derivatization.

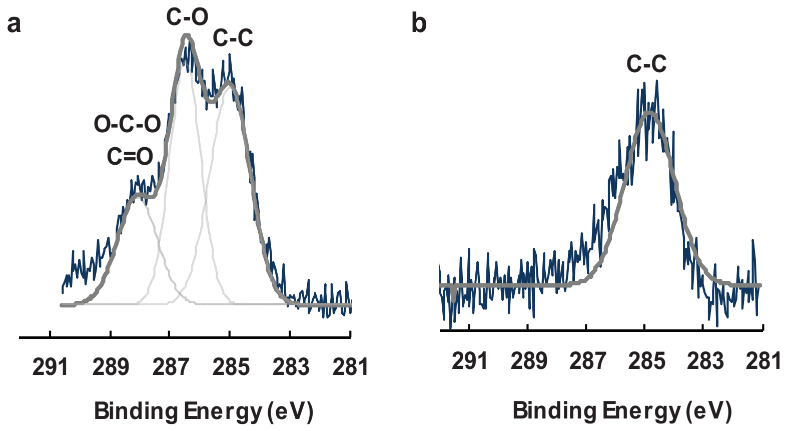

Figure S2. High-resolution XPS spectra of (**a**) GlcNAc6-immobilized and (**b**) GlcNAc6-free Au surfaces.

© 2016 by the authors; licensee MDPI, Basel, Switzerland. This article is an open access article distributed under the terms and conditions of the Creative Commons by Attribution (CC-BY) license (http://creativecommons.org/licenses/by/4.0/).
